# The impact of genetic diversity on the accuracy of DNA barcoding to identify species: A study on the genus *Phellodendron*

**DOI:** 10.1002/ece3.5590

**Published:** 2019-08-20

**Authors:** Zhi‐peng Zhang, Xiao‐yue Wang, Zhao Zhang, Hui Yao, Xiao‐mei Zhang, Yang Zhang, Ben‐gang Zhang

**Affiliations:** ^1^ Key Laboratory of Bioactive Substances and Resources Utilization of Chinese Herbal Medicine Ministry of Education/Institute of Medicinal Plant Development Chinese Academy of Medical Sciences Peking Union Medical College Beijing China; ^2^ China‐ASEAN Traditional Medicine Cooperation and Exchange Center Guangxi Botanical Garden of Medicinal Plants Nanning China

**Keywords:** accuracy, DNA barcoding, genetic diversity, identify species, *Phellodendron*

## Abstract

DNA barcoding is widely used in species identification, but there is considerable controversy regarding the extent of sampling in research methods. Some scholars have proposed that this small sample size underestimates the intraspecific genetic diversity, which would impact on the accuracy of DNA barcoding to identify species. In study, we selected all *Phellodendron* species (including *P. amurense* Rupr., *P. chinense* Schneid., and *P. chinense* var. *glabriusculum* Schneid.) as the materials, collected 59 *P. amurense* samples from 35 populations greatly to represent the genetic diversity, and analyzed the haplotype, genetic distance, barcoding gap, and Neighbor‐Joining (NJ) trees based on *psb*A*‐trn*H and internal transcribed spacer gene sequences. Additionally, a sampling simulation was conducted to assess the correlation between genetic diversity and the number of populations. Finally, analysis of critical geographical populations was performed. Based on analysis of haplotype, genetic distance, barcoding gap, and NJ trees, we found that eight *P. amurense* samples impacted on the effectiveness of DNA barcoding, which genetic information were very important to identify *Phellodendron* species. Moreover, the result of the NJ tree analysis performed the small‐scale *P. amurense* sample size did not completely match the objective phylogenetic relationship in *Phellodendron*. In simulation sampling analysis, the data showed the genetic diversity indexes at the same population level gradually decreased and stabilized as the number of simulation sampling populations increased. We found that 1–2 samples from over 24 populations based on uniform geographical distribution could represent 80% of the genetic diversity of *P. amurense* and ensure authenticity and reliability of DNA barcoding. Thus, we proposed it is particularly important adequately samples to cover infraspecific genetic diversity in order to ensure identification accuracy of DNA barcoding.

## Graphical abstract

We proposed adequately covering infraspecific variation should be focused more on establishment databases and application of DNA barcode. Small‐scale samples for each species may be not enough to represent the genetic diversity of the species and had a huge risk in application of DNA barcode.

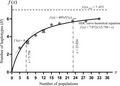

**Highlights**

- Used a case to prove the genetic diversity impacting on the identification accuracy of DNA barcoding.
- Analyzed the relationship between samples size and genetic diversity parameters of *P. amurense* by simulation sampling.
- Proposed adequately samples covering infraspecific variation of species being the key to DNA barcoding.

## 1 INTRODUCTION

DNA barcoding, which is based on one or more common and standard DNA sequences for species identification and characterization techniques, is widely used in the survey and inventory of biodiversity, species identification, and discovery of new species (Hebert, Cywinska, Ball, & Dewaard, 2003; HollingsworthGraham & Little, 2011; Kress, Wurdack, Zimmer, Weigt, & Janzen, 2005). Cytochrome oxidase I gene (*CO*I gene) sequences from the mitochondrial genome can be used for the identification of multiple animal groups, making it an ideal animal barcode sequence (Tavares & Baker, 2008; Ward, Zemlak, Innes, Last, & Hebert, 2005). In terms of plants, no one barcode can be a universal barcode similar to the animal *CO*I gene. The Consortium for the Barcode of Life used the ribulose bisphosphate carboxylase large chain (*rbc*L) and maturase K (*mat*K) genes as core DNA barcodes for seed plants, as well as the *psb*A*‐trn*H intergenic spacer (*psb*A*‐trn*H) and internal transcribed spacer (ITS) genes as supplementary DNA barcodes (CBOL Plant Working Group, 2009). Since Hebert proposed the concept of DNA barcodes in 2003 (Hebert et al., 2003), many scholars have performed studies to verify the feasibility of identifying plant species through DNA barcoding. The China Plant BOL Group (2011) assessed *rbc*L, *mat*K, *psb*A*‐trn*H, and ITS candidate barcodes with 75 families, 141 genuses, 1,757 species, and 6,286 samples from seed plants. Many studies have further confirmed the effectiveness of DNA barcoding for species identification. For example, Yao, Song, and Ma (2009) reported that the *psb*A*‐trn*H sequence can be used to identify *Dendrobium* species. Liu, Zhang, et al. (2012) showed a 100% identification using the *psb*A*‐trn*H sequence in 38 species from the genus *Rhododendron*. Li, Chen, Wang, and Xiong (2012) discovered that the efficiency of the ITS sequence is 70% when examining 63 species in the genus *Ficus*. Yang, Wang, Möller, Gao, and Wu (2012) used ITS + *psb*A*‐trn*H sequences to identify 30 species from the genus *Parnassia* with 90% efficiency.

However, the genetic diversity of each species was neglected by almost all DNA barcoding studies, which reflected in sampling strategies. The International Barcode of Life Project calls for at least 10 samples for each species (Gao et al., 2012). To obtain more species sequences, one must decrease the number samples for each species when there is limited funding (Matz & Nielsen, 2005). Currently, most species have only 5–10 sequences in the established DNA barcode database, though several species have only 1–2 sequences (http://www.barcodinglife.org/views/ligin.php), which is far from sufficient to speculate the number of samples needed to establish a database to represent the genetic diversity of each species. The efficiency and accuracy of DNA barcoding depends on the degree of sampling per species, because a large enough sample size is needed to provide a reliable estimate of genetic polymorphism and for delimiting species (Luo et al., 2015). Incomplete sample surveys, errors in sample identification and weak taxonomic are obstacles (Young, McKelvey, Pilgrim, & Schwartz, 2013). A small range and unevenness in sampling could cause differences in thresholding between intraspecific and interspecific variations (Meyer & Paulay, 2005). We believed that mostly representation of genetic variation in samples had defects, as many studies only analyze and collect 1 or 2 samples from one species. Therefore, we chose *Phellodendron* genus as a case to discuss genetic diversity impacting on the accuracy of DNA barcoding to identify species.

The *Phellodendron* genus (Rutaceae) has two species and one variant, including *P. amurense* Rupr., *P. chinense* Schneid., and *P. chinense* var.* glabriusculum* Schneid., are distributed in China (Huang, 1997). As tertiary paleotropical flora relict plants, *Phellodendron* has scientific value for studying ancient flora, paleogeography, and quaternary glacial climate (Huang, 1958). A large number of wild populations drastically reduced, especially since the late 19th century, because its cortex is a kind of precious Chinese traditional medicine, and its wood is widely used for its hard texture and beautiful grain and color (Qin, Wang, & Yan, 2006). The endangered plant *P. amurense* is distributed in northeastern China, and *P. chinense* is distributed in southwestern China (Huang, 1997; State Bureau of Environmental Protection of China & Institute of Botany Chinese Academy of Sciences, 1987); the gap in their distribution area is approximately 1,000 km. These genetic studies mainly focused on intraspecific genetic diversity, the analyses of intraspecies and intergenus have not been reported (Wang, Bao, Wang, & Ge, 2014; Yan, Zhang, Zhang, & Yu, 2006; Yu et al., 2013). *Phellodendron* genus does not exist controversy in plant taxonomy and have the geographical isolation of species. These studies mainly focused on intraspecific genetic diversity and concerned the species in China; however, analyses of intraspecies and intergenus have not been reported in the genus *Phellodendron*. Thus, it is an ideal material. To assess the impact of genetic diversity on DNA barcoding, we selected the *Phellodendron* genus as a model and collected numerous samples to represent intraspecific diversity in study.

Also, the data of previous study showed that there was no variation of *rbc*L, *mat*K among individuals of *Phellodendron*, *psb*A*‐trn*H and ITS were polymorphism in *Phellodendron*. Thus, we assessed the accuracy of DNA barcoding to determine *Phellodendron* species by ITS and *psb*A*‐trn*H.

## 2 MATERIALS AND METHODS

### 2.1 Plant materials

We collected 1 or 2 samples from each population, which based on a viewpoint that DNA barcoding variations within a population are usually less than that between populations (Liu, Provan, Gao, & Li, 2012). In total, 59 *P. amurense* cortex samples were densely collected from 35 populations throughout the distribution area, which enabled us to have a large size and representative samples to ensure the credibility of this study.

**Figure 1:**
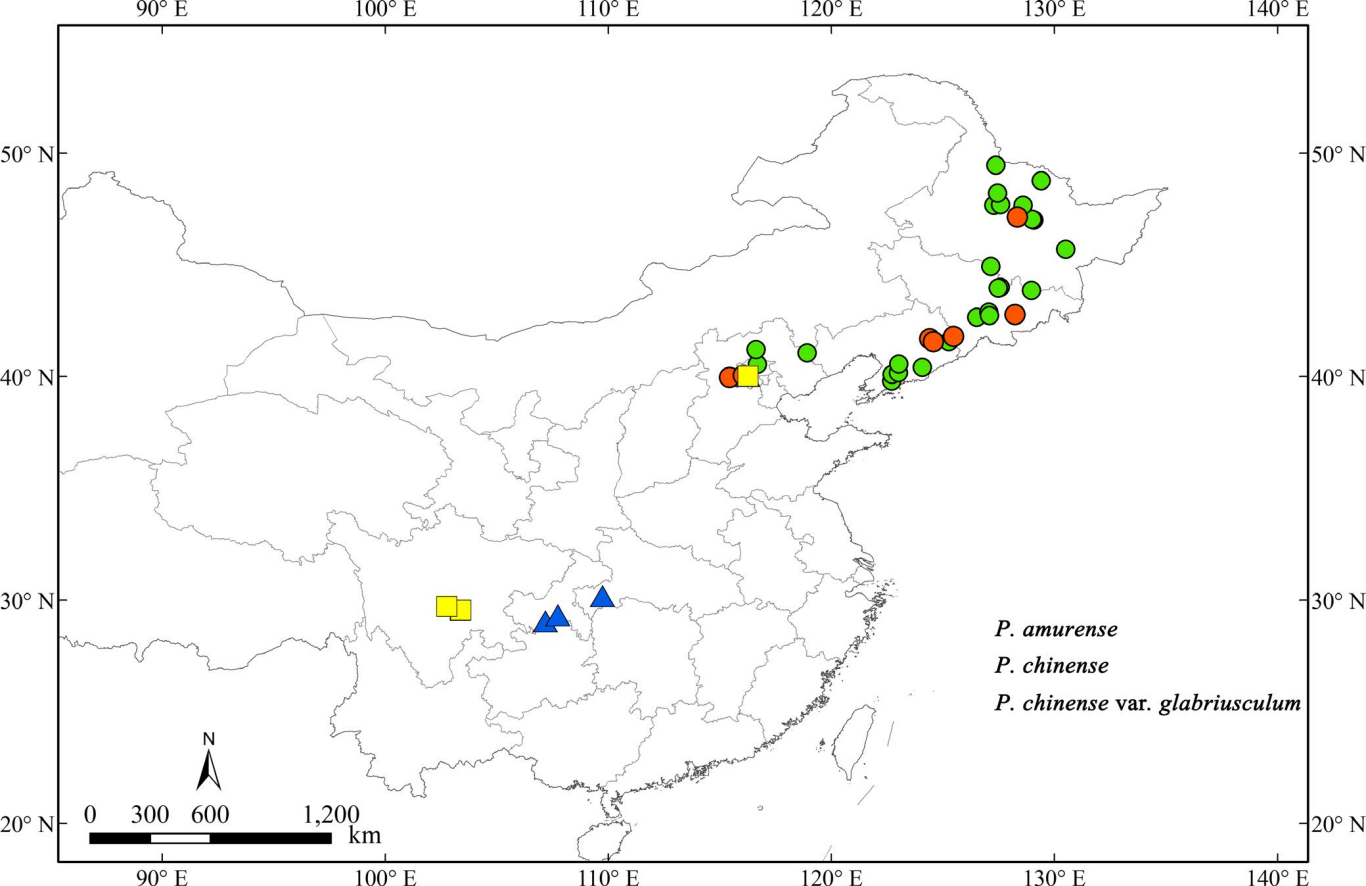
Geographical distribution of 41 sample populations (red circles mark critical populations)

The survey found that *P. chinense* and *P. chinense *var.* glabriusculum* populations included types that were both were rare and wild, as well as cultivated; thus, we collected one wild population from *P. chinense* and *P. chinense *var.* glabriusculum*. Fourteen *P. chinense* and *P. chinense *var.* glabriusculum* samples were collected from eight populations to ensure the samples represent the entire distribution area. A total of 41 populations were surveyed and are shown in Figure 1.

Additionally, four samples of *Tetradium ruticarpum* were collected as an outgroup for the molecular analysis. The significant differences in the geographical distribution between *P. amurense* and other *Phellodendron* species would completely guarantee the accuracy of the classification. Although five samples from three plants were collected from a botanical garden, detailed records of their original location provided evidence to ensure their identity. The botanical identities of all the samples were confirmed by Professor Zhao Zhang. All corresponding voucher samples were deposited in the Herbarium of the Institute of Medicinal Plant Development.

### 2.2 DNA extraction and amplification

All samples (40 mg) were rubbed for 2 min at a frequency of 30 r/s. Total genomic DNA was extracted using the Plant Genomic DNA Kit (Tiangen Biotech Co.) according to the manufacturer's instructions. The extracted genomic DNA was amplified by polymerase chain reaction (PCR) using the ITS (ITS5F and ITS4R) and *psb*A*‐trn*H (fwdPA and revTH) primers (Chen, 2012). The PCR mixtures and conditions were described by Chen (2012). PCR products were separated and detected by 1.5% agarose gel electrophoresis. Purified products were sequenced in both directions using the PCR primers on a 3730XL sequencer (Applied Biosystems).

### 2.3 Statistical analysis

Sequences were assembled and aligned with the CodonCode Aligner 3.7.1 (CodonCode Co.) as well as the base quality was evaluated to avoid technical error. The inter/intraspecific genetic distances and barcoding gap were analyzed with the *P* Language based on Kimuraʼs 2‐parameter theory (Tamura et al., 2011). The variable sites and the bootstrap Neighbor‐Joining (NJ) tree were conducted with MEGA (4.0 version) according to Kimuraʼs 2‐parameter method with 1,000 replicate bootstrap testing (Tamura et al., 2011).

To assess the relationship between a number of populations and the genetic diversity of *P. amurense*, a sampling simulation was conducted in this study. In total, 3, 6, 9, 12, 15, 18, 23, 28, and 33 populations levels were randomly selected from 35 *P. amurense* population samples by a computer sampling simulation, with each population sampling being repeated 50 times. The number of haplotypes (*H*) was used as an important indicator of genetic diversity in studies of populations calculated by the MEGA (4.0 version) (Tamura et al., 2011) and DnaSP V5.0 (Librado & Rozas, 2009) software. The haplotype diversity (*H*_d_) and nucleotide diversity (*P_i_*) were also studied at the same time. The haplotype diversity (*H*_d_), the frequencies of two different haplotypes randomly extracted in the sample, is an important indicator of the degree of variation in a population. The nucleotide diversity (*P_i_*), the average of all pairwise distances in a sample, is commonly used to estimate genetic polymorphism. The relationship of *H* with a population's number in the sampling simulation was analyzed by nonlinear regression (curve fit) with GraphPad Prism 5 (https://www.graphpad.com/), while the relationships of numbers of populations with *H*_d_ and *P_i_* were analyzed with a scatter plot.

## 3 RESULTS

### 3.1 Haplotype analysis

The GenBank accession No. for the ITS and *psb*A*‐trn*H contig sequences from all the samples in this study are shown in Table 1. The average base quality value (QV) of forward sequence or reverse sequence was ≥30, and the coincidence ratio of forward and reverse sequences was 100%. The haplotype and variable sites in the ITS sequence are shown in Table 2. The quality value (QV) of variable sites was verified as ≥30 by traceability. The data revealed that *P. amurense* had 6 haplotypes, *P. chinense* had four haplotypes, and *P. chinense *var.* glabriusculum* had three haplotypes. *P. chinense* was different from *P. amurense* and *P. chinense *var.* glabriusculum* at bp 173 with a T; simultaneously *P. chinense *var.* glabriusculum* was also different from *P. amurense* and *P. chinense* at bp 208 with a T, except in haplotypes A12 and A13.

**Table 1:** GenBank accession No. for the samples in this study

Codes	Scientific name	Family	GenBank accession No.	
			ITS	*psb*A‐*trn*H
A1‐A59	*P. amurense*	Rutaceae	MK419239–MK419297	MK419162–MK41922
B1‐B6	*P. chinense*	Rutaceae	MK419298–MK419303	MK419221–MK419226
C1‐C8	*P. chinense* var*. glabriusculum*	Rutaceae	MK419304–MK419311	MK419227–MK419234
D1‐D4	*Tetradium ruticarpum*	Rutaceae	MK419312–MK419315	MK419235–MK419238

**Table 2:** ITS sequence haplotypes and variation sites in *Phellodendron* species

Scientific name	Haplotype	Number of samples	Percentage	Sites/bp											
				1	1	1	1	2	3	4	4	4	4	5	6
				5	7	7	9	0	9	1	3	4	8	9	2
				7	3	7	0	8	1	9	5	3	6	9	3
*P. amurense*	A1	50	84.7	C	C	G	G	C	C	C	C	C	C	G	A
	A2	3	5.1	.	.	.	.	.	T	.	.	.	.	.	.
	A3	1	1.7	.	.	.	A	.	.	.	.	.	.	.	.
	A4	1	1.7	.	.	.	.	.	.	G	.	.	.	.	.
	A5	1	1.7	.	.	.	.	.	.	.	.	.	.	.	C
	A6	3	5.1	.	.	A	.	.	.	.	.	.	.	.	.
*P. chinense*	A7	2	33.3	.	T	.	.	.	.	.	.	.	.	A	.
	A8	2	33.3	.	T	.	.	.	.	.	.	.	.	.	.
	A9	1	16.7	.	T	.	.	.	.	.	.	.	T	.	.
	A10	1	16.7	T	T	.	.	.	.	.	.	.	.	.	.
*P. chinense* var. *glabriusculum*	A11	6	75	.	.	.	.	T	.	.	.	.	.	.	.
	A12	1	12.5	.	.	.	.	.	.	.	.	A	.	.	.
	A13	1	12.5	.	.	.	.	.	.	.	T	A	.	.	.

(.) indicated the same base as the first row.

The haplotype and variable sites in the *psb*A*‐trn*H sequence (Table 3), which the quality value (QV) of variable sites, were verified as ≥30 by traceability, demonstrated that *P. chinense* and *P. chinense *var.* glabriusculum* had one haplotype (B10) in common, and that *P. amurense* had nine haplotypes, with 54.1% of the samples being haplotype B1. Based on the variable sites, haplotypes B2 and B3 in *P. amurense* were the same as haplotype B10 with 379, 380, 381, and 382 bp.

**Table 3:** *psb*A*‐trn*H sequence haplotypes and variation sites in *Phellodendron* species

Scientific name	Haplotype	Number of samples	Percentage	Sites/ bp										
				2	3	1	5	2	3	3	3	3	3	4
								0	7	8	8	8	9	4
						4	9	6	9	0	1	2	7	5
*P. amurense*	B1	32	54.1	G	C	C	A	T	T	T	G	C	A	T
	B2	5	8.5	.	.	.	.	.	G	C	A	A	.	.
	B3	3	5.1	.	.	T	.	.	G	C	A	A	.	.
	B4	13	22.1	.	.	T	.	.	.	.	.	.	.	.
	B5	1	1.7	C	.	T	.	.	.	.	.	.	.	.
	B6	2	3.4	C	.	.	C	.	.	.	.	.	.	.
	B7	1	1.7	.	A	.	.	.	.	.	.	.	.	.
	B8	1	1.7	‐	G	T	.	.	.	.	.	.	.	.
	B9	1	1.7	.	.	.	.	.	.	.	.	.	.	C
*P. chinense*	B10	6	100	.	.	.	.	G	G	C	A	A	G	.
*P. chinense* var. *glabriusculum*	B10	8	100	.	.	.	.	G	G	C	A	A	G	.

(.) indicated the same base as the first row.

### 3.2 Genetic distance and barcoding gap analysis

Six parameters were used to analyze intraspecific variation and interspecific divergence with two barcodes (Table 4). In this instance, the maximum intraspecific distance was higher than the minimum interspecific distance for two barcodes, which indicated that the two barcodes did not perform well in the discrimination of *Phellodendron* species.

**Table 4:** Analysis of the intergenus‐specific divergence and intraspecific variation with the two barcodes

Markers	ITS	*psb*A*‐trn*H
Theta(avg_intra_avg)	0.0018 ± 0.0020	0.0018 ± 0.0026
coalescent depth(avg_intra_max)	0.0048 ± 0.0023	0.0081 ± 0.0115
All intraspecific distance(avg_between_intraspecies)	0.0005 ± 0.0010	0.0035 ± 0.0041
Theta prime(avg_interbyG_avg)	0.0024 ± 0.0010	0.0137 ± 0.0041
minimum interspecific distance(avg_interbyG_min)	0.0016 ± 0.0010	0.0046 ± 0.0041
all interspecific distance	0.0024 ± 0.0009	0.0137 ± 0.0036

**Figure 2:**
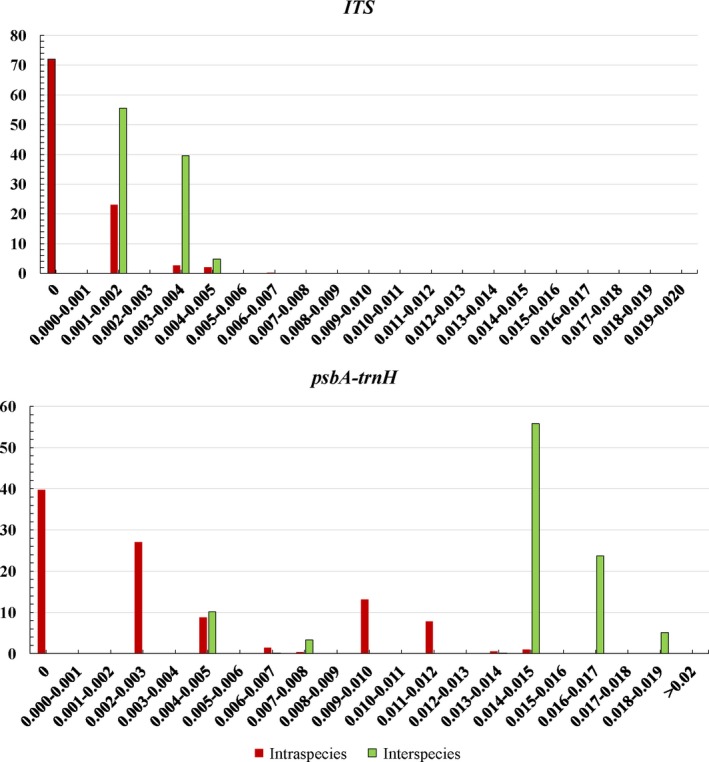
Relative distribution of the interspecific and intraspecific variation using the two barcodes based on the K2P genetic distance

The barcoding gap presents the remarkable variation between inter‐ and intraspecies and demonstrates the separate or overlapping distributions between intra‐ and interspecific samples. In this study (Figure 2), the ITS sequence did not exhibit gaps in the intra‐ and interspecific variation distributions. In contrast, the *psb*A*‐trn*H sequence displayed murky barcoding gaps with overlapping intra‐ and interspecific variation distributions. Through calculation and traceability, we found that eight *P. amurense* samples had overlapping regions with haplotypes B2 and B3.

### 3.3 NJ tree analysis

**Figure 3:**
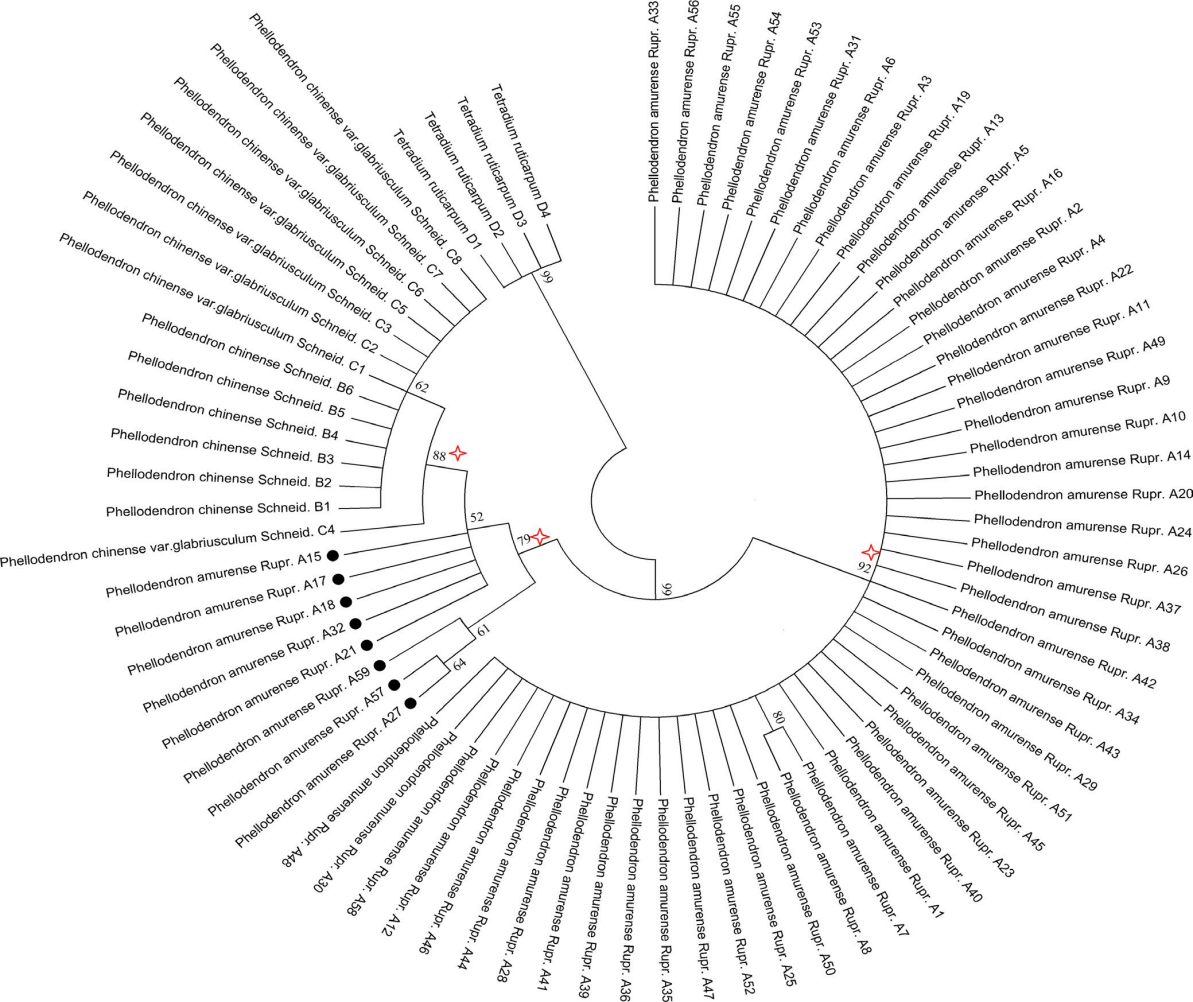
*Phellodendron* species NJ tree with the *psb*A‐*trn*H sequence (The bootstrap scores [1,000 replicates] are shown for each branch)

An NJ tree illustrates the relationship among species and facilitates their clustering. In this study, DNA barcode NJ trees were built based on the K2P model. A NJ tree built on the ITS sequence demonstrated that *P. amurense*, *P. chinense,* and *P. chinense* var*. glabriusculum* were short of monophyly. The NJ tree built on the *psb*A*‐trn*H sequences clustered into two major branches (Figure 3), with 86.4% of *P. amurense* clustering into one branch, and the other eight samples clustering with *P. chinense* and *P. chinense* var*. glabriusculum* into the other branch. This finding meant that the phylogenetic relationship of *P. amurense* with *P. chinense* and *P. chinense* var*. glabriusculum* was paraphyly. Furthermore, eight *P. amurense* samples were particularly important for their intraspecific genetic variation and for species identification; these samples were haplotypes B2 and B3 by traceability.

**Figure 4:**
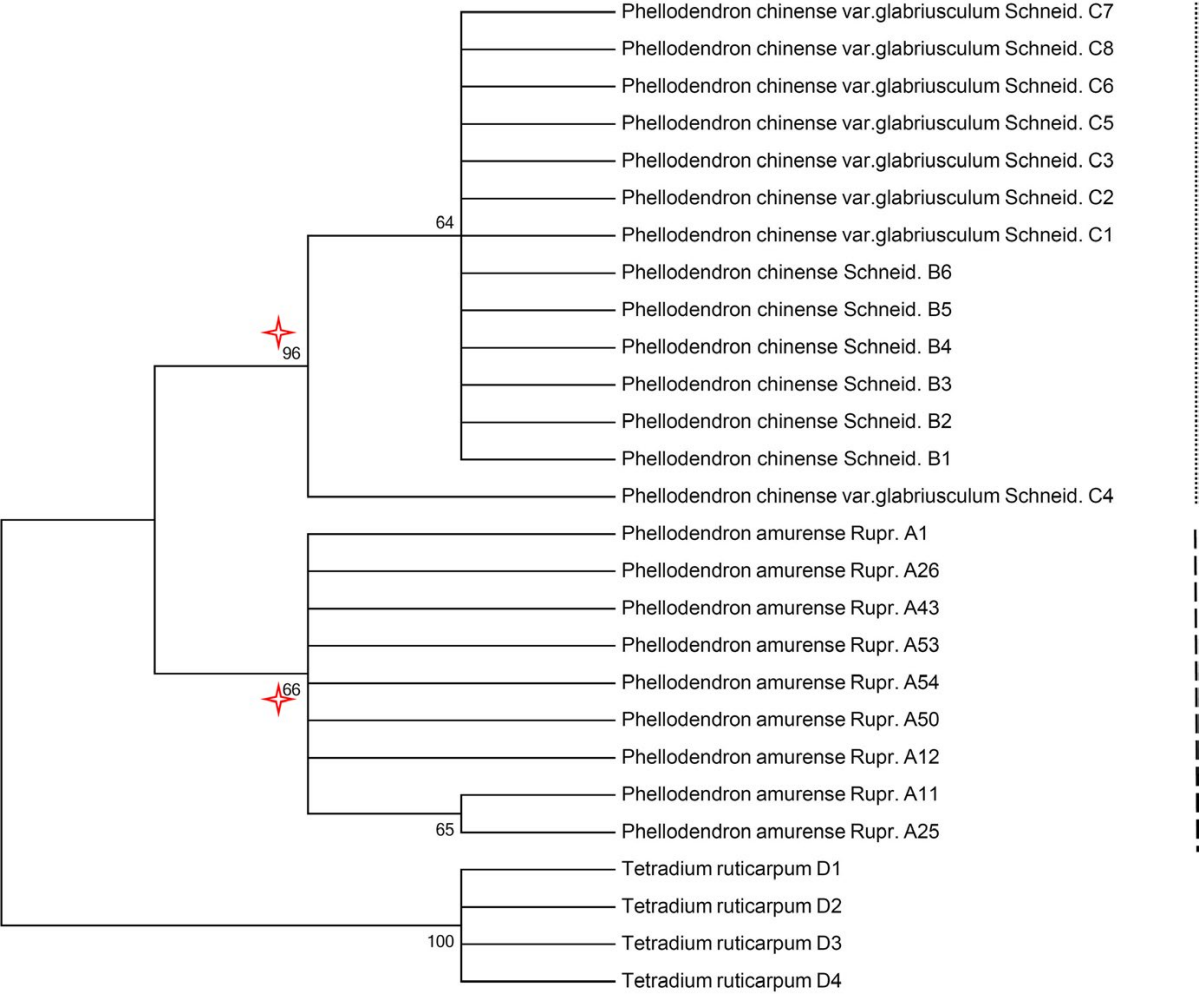
Typical sample NJ tree pattern for reciprocal monophyly based on small‐scale *P. amurense* with the *psb*A‐*trn*H sequence (The bootstrap scores [1,000 replicates] is shown for each branch)

**Figure 5:**
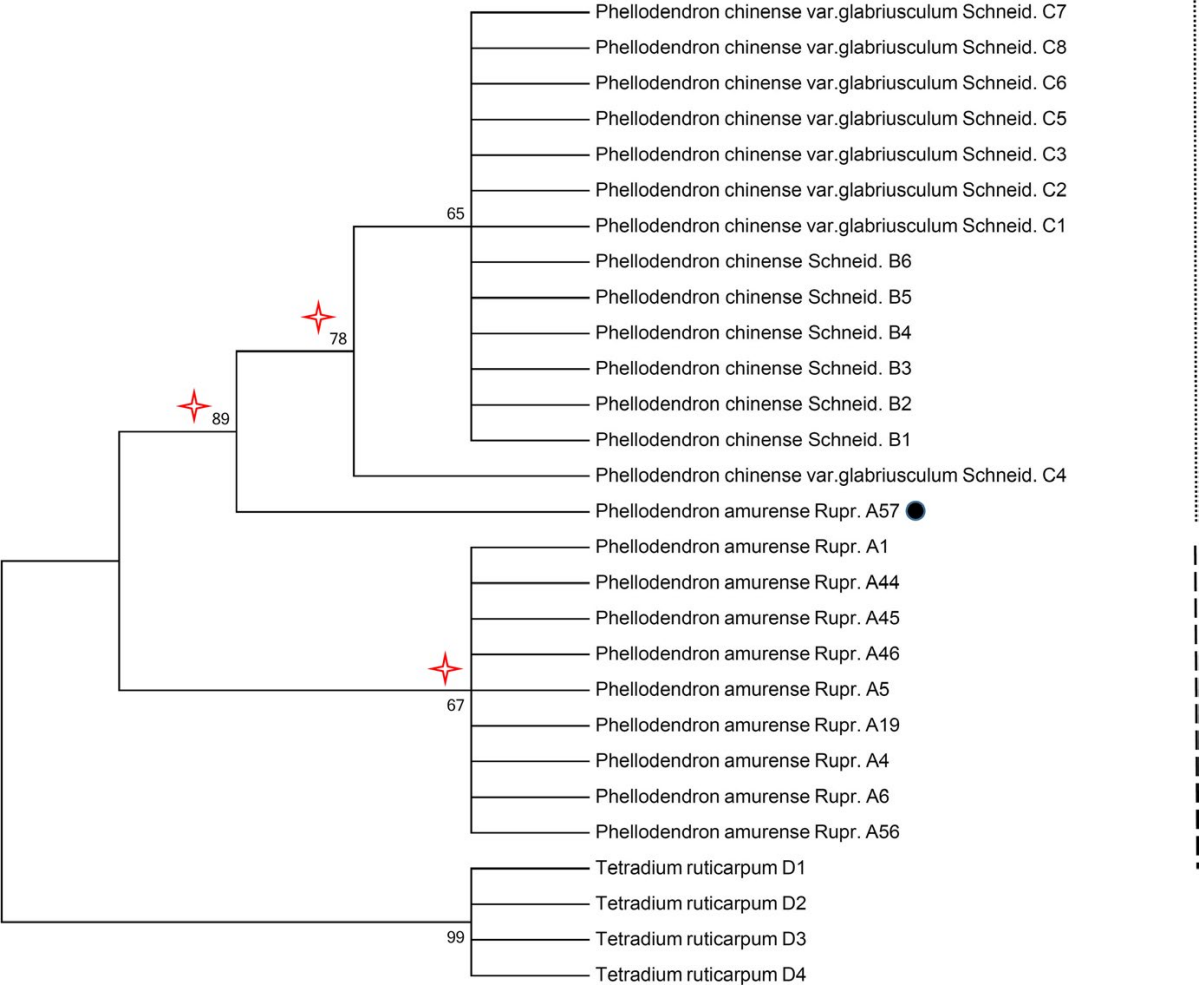
Typical NJ tree pattern of paraphyly based on small‐scale *P. amurense* samples with the *psb*A‐*trn*H sequence (The bootstrap scores [1,000 replicates] are shown for each branch)

When we performed the NJ tree analysis using a small‐scale randomly selected samples from *P. amurense*, two typical NJ tree patterns existed. One pattern was the reciprocal monophyly in Figure 4, in which members from *P. amurense* and other species shared a unique common ancestor. The other was paraphyly (Figure 5), in which the *P. amurense* species is monophyletic but nests within another recognized species. Therefore, the adequately samples to cover infraspecific variation is essential for DNA barcoding.

### 3.4 Genetic diversity parameters in the simulation sampling analysis

**Figure 6:**
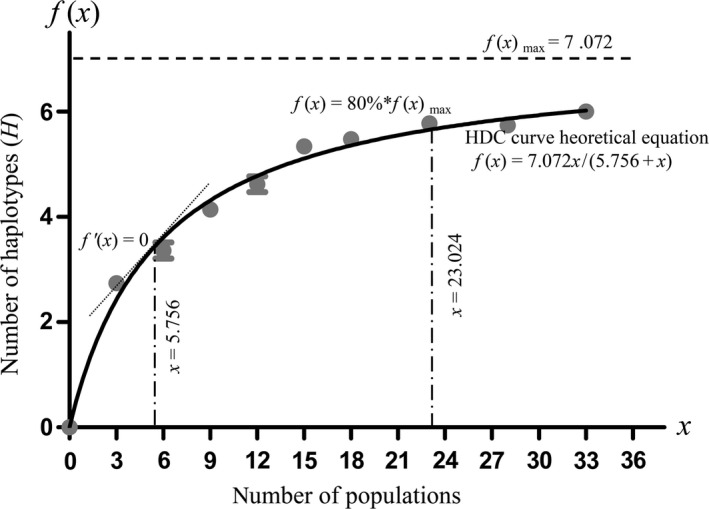
Haplotype number versus population number curve

**Figure 7:**
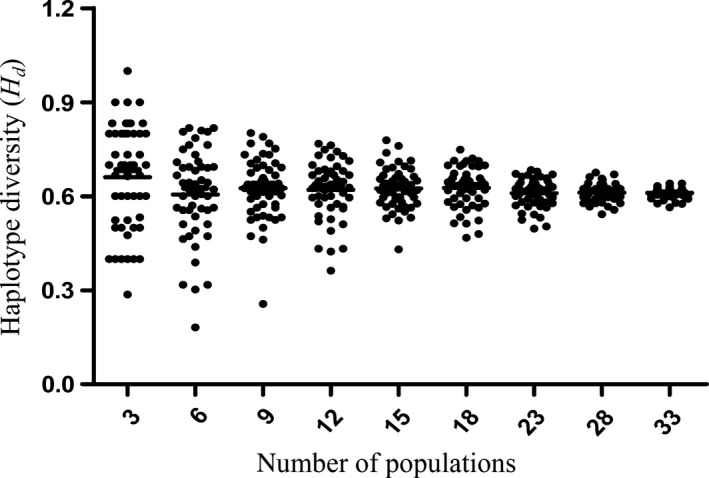
Scatter plot of haplotype diversity

**Figure 8:**
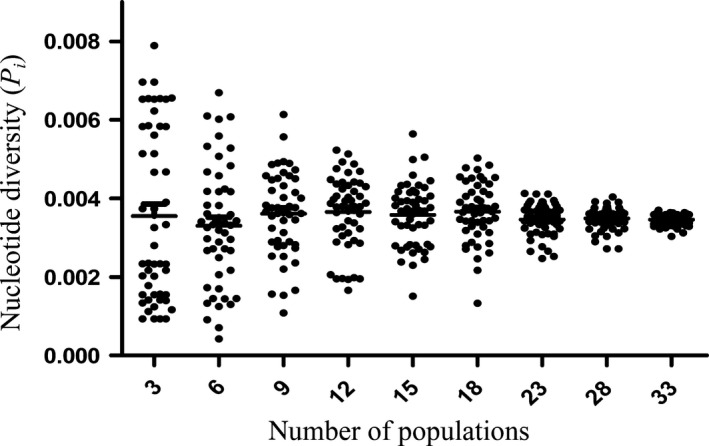
Scatter plot of nucleotide diversity

Since *psb*A*‐trn*H performed better than ITS for identifying the *Phellodendron* species, we analyzed the relationship between the number of populations and the genetic diversity parameters based on *psb*A*‐trn*H for *P. amurense*. The Michaelis–Menten equation [*f*(*x*) = a*x*/(b + *x*)] is used to perform nonlinear regression (curve fit), which satisfied the requirements in this study. The haplotype discovery curve (HDC) is presented in Figure 6 with the theoretical equation *f*(*x*) = 7.072*x*/(5.756 + *x*) and an *r* ^2^ = .8082. The results showed that the number of haplotypes (*H*) index gradually increased with the increase in the simulation sampling populations and had an overall sample level as shown in Figure 6. The scatter plots of the population number with haplotype diversity (*H*_d_) and nucleotide diversity (*P_i_*) are shown in Figures 7 and 8, respectively. These plots explained that the dispersion of the genetic diversity index at the same population level gradually decreased and stabilized as the number of simulation sampling populations increased.

### 3.5 Theoretical key number of sample sizes in the simulation sampling analysis

The number of haplotypes (*H*) was a pivotal criterion for estimating the genetic diversity of *P. amurense* in simulation sampling. We arrived at the haplotype discovery curve (HDC) in Figure 6 with the theoretical equation *f*(*x*) = 7.072*x*/(5.756 + *x*). We focused on the following two theoretical key parameters for sample sizes in this study: (a) the threshold of sample sizes where new haplotypes become considerably more difficult to identify with extra sampling efforts (the first‐order derivative of the HDC curve is equal to zero) and (b) the sample size that includes the majority of haplotypes (indicating that 80% of haplotypes are found).

The theoretical key parameters are presented in Figure 6 after careful calculation based on the HDC curve theoretical equation. The figure showed that the theoretical threshold of the sample size is 5.756, which meant that discovering a new haplotype is much harder with only six populations in the simulation sampling. Furthermore, we could obtain 80% of *P. amurense* haplotypes when the theoretical threshold of the sample size was 23.024 populations using simulated sampling with the theoretical equation or sample sizes with no less than 24 populations in an actual sampling. To obtain a higher percentage of *P. amurense* haplotypes, we should increase the sample size.

### 3.6 Critical analysis on populations

Since *psb*A‐*trn*H performed better than ITS for identifying *Phellodendron* species, we analyzed the key populations based on *psb*A‐*trn*H. Combining the haplotype analysis with the NJ tree analysis, we discovered eight special samples with seven populations existing in all the *P. amurense* populations. The samples' variable sites and genetic relationship were similar to *P. chinense* and *P. chinense* var*. glabriusculum* based on our data (Table 3 and Figure 3). Therefore, missing these *P. amurense* sample haplotypes would directly affect the reliability of sampling research results. We defined the eight *P. amurense* samples as critical samples and their populations as critical populations based on sufficient evidence. The geographical distribution of the seven critical *P. amurense* populations is shown in Figure 1 as red circles. We observed that they were uniformly distributed in the whole *P. amurense* area. This meant there was no key geographical group of *P. amurense*. Thus, it is highly meaningful to assess the sampling size from the uniform geographical distribution based on the genetic diversity.

## 4 DISCUSSION

It is fact that different plant species will vary in the amount of infraspecific variation they contain, because of variation in length of time since divergence, and variation. Speciation is a gradual process, meaning that the discussion of species based on the evolutionary history of a cross section has significant limitations (Hennig, 1966). Intraspecific genetic variation of species is not only an integral part of evolutionary history but also changes continuously. Objectively, each species possesses abundant intraspecific genetic variation, and the intraspecific variation of ITS and *psb*A‐*trn*H sequences is natural. It is necessary to assess the intraspecific genetic diversity of species. Furthermore, lack the genetic information of key populations will lead to DNA barcoding gaps, and the number of samples for collection is crucial to establish a reliable reference database for species identification (Meyer & Paulay, 2005; Wiemer & Fiedler, 2007). Dasmahapatra and Mallet (2006) believes that many studies only analyze and collect 1 or 2 samples from one species within a limited geographical scope, which seriously underestimates the intraspecific variation and may lead to false‐positive results.

In study, we paid close attention to the genetic diversity of *P. amurense* and collected adequately samples throughout the distribution area to ensure covering infraspecific variation. We conducted a comparative analysis of the overall samples and small‐scale randomly samples. The results showed that small‐scale samples could not demonstrate an objective phylogenetic relationship in *Phellodendron* (Figures 3, 4 and 5). It meant that an insufficient sample would reduce identification accuracy of the DNA barcoding. In the simulation sample analysis, we found 1–2 samples from 23 populations based on uniformed geographical distribution are necessary to obtain critical samples and represent 80% of the genetic diversity of *P. amurense* (Figure 6). It did not support other studies, such as Liu, Provan, et al. (2012) believe that 8–10 samples of each species are representative of the genetic diversity in *Taxus*, and Matz and Nielsen (2005) recommended that 12 samples from a species can used to study DNA barcoding. We agreed with Zhang, He, Crozier, Muster, and Zhu (2010) hold that 5–10 samples for each species are not sufficient to represent species genetic diversity. When studying DNA barcoding with closely related species, obtaining a specific sample size for each species should become the research objective. Our research showed that errors may occur in the DNA barcoding identification method, which would establish unrepresentative samples sizes, including the lack of the B2 and B3 haplotypes. The key geographical populations have a great impact on the accuracy of DNA barcoding to identify species (Bergsten et al., 2012; Meyer & Paulay, 2005). The B2 and B3 haplotype populations were uniformly distributed in the entire *P. amurense* area, which meant there were no key geographical groups (Figure 1). *P. amurense* clearly underwent genetic variation among the existing populations. It was successful to take large‐scale uniform sampling covering the entire distribution area in study.

The most important challenge for species identification is DNA barcodes used for closely related species and recently differentiated species (Newmaster, Fazekas, Steeves, & Janovec, 2008). DNA barcoding has been applied to crude drug identification (Kool et al., 2012; Techen, Parveen, Pan, & Khan, 2014), but we had to keep doubts about the identification accuracy of DNA barcoding based on the results of our study. Whether the sample size of each species may represent the actual levels of genetic diversity in the current database needs to be studied (Chen, 2015; Chen et al., 2014). In addition, the lack of key plant specimens will lead to narrow genetic levels in the database resulted in DNA barcoding failures. Furthermore, the input and output ratio is an important factor to restrict large sample strategies implemented in DNA barcoding database of each species.

In order to ensure identification accuracy of DNA barcoding which is used as tools for species identification, it is particularly important to collect adequately samples covering infraspecific genetic diversity of species.

## CONFLICT OF INTEREST

The authors declare no conflicts of interest.

## AUTHORS CONTRIBUTIONS

Zhi‐peng Zhang: experimental operation, manuscript writing; Xiao‐yue Wang: experimental operation; Zhao Zhang: corresponding author, experimental design; Hui Yao: manuscript modification; Xiao‐mei Zhang: manuscript modification; Yang Zhang: sample Collection; Ben‐gang Zhang: experimental design.

## Data Availability

ITS and *psb*A*‐trn*H sequence data can be accessed in GenBank. The GenBank accession No. is shown in Table 1.
